# Effects of dapagliflozin on myoglobin efflux from cardiomyocyte during myocardial ischemia/reperfusion in anesthetized rats

**DOI:** 10.1038/s41598-024-67195-3

**Published:** 2024-07-16

**Authors:** Tomohiro Hayashida, Yosuke Kuroko, Shuji Shimizu, Tsuyoshi Akiyama, Takanori Suezawa, Yukio Kioka, Yasuhiro Kotani, Toshiaki Shishido, Shingo Kasahara

**Affiliations:** 1 Department of Cardiovascular Surgery, Okayama University Graduate School of Medicine, Dentistry and Pharmaceutical Sciences, Okayama, 700-8558 Japan; 2 Department of Cardiovascular Surgery, Fukuyama City Hospital, Fukuyama, Hiroshima 721-8511 Japan; 3 Department of Cardiovascular Surgery, Okayama University Graduate School of Medicine, Dentistry and Pharmaceutical Sciences and Okayama University Hospital, Okayama, 700-8558 Japan; 4 Department of Research Promotion and Management, National Cerebral and Cardiovascular Center, 6-1 Kishibe-Shimmachi, Suita, Osaka 564-8565 Japan; 5 Department of Cardiac Physiology, National Cerebral and Cardiovascular Center, Suita, Osaka 564-8565 Japan

**Keywords:** Sodium-glucose-cotransporter 2 inhibitor, Dapagliflozin, Myocardial ischemia/reperfusion, Cardiac microdialysis, Myoglobin, Physiology, Circulation

## Abstract

It has been suggested that sodium-glucose cotransporter 2 (SGLT2) inhibitors have cardioprotective effects during myocardial ischemia/reperfusion (I/R) independent of glucose-lowering action. However, the effects of SGLT2 inhibitors on structural damage to cardiomyocytes in the ischemic region during I/R remain unknown. We applied a microdialysis technique to the heart of anesthetized rats and investigated the effects of an SGLT2 inhibitor, dapagliflozin, on myocardial interstitial myoglobin levels in the ischemic region during coronary occlusion followed by reperfusion. Dapagliflozin was administered systemically (40 μg/body iv) or locally via a dialysis probe (100 μM and 1 mM) 30 min before coronary occlusion. In the vehicle group, coronary occlusion increased the dialysate myoglobin concentration in the ischemic region. Reperfusion further increased the dialysate myoglobin concentration. Intravenous administration of dapagliflozin reduced dialysate myoglobin concentration during ischemia and at 0–15 min after reperfusion, but local administration (100 μM and 1 mM) did not. Therefore, acute systemic administration of dapagliflozin prior to ischemia has cardioprotective effects on structural damage during I/R.

## Introduction

Several studies have already reported the cardioprotective effects of SGLT2 inhibitors in ischemic myocardium^1^. Andreadou et al. have reported that SGLT2 inhibitors mitigate acute myocardial ischemia/reperfusion (I/R) injury by attenuating cardiac infarct size, improving left ventricular function, and reducing arrhythmias^2^. They have also demonstrated that in the early stage of acute myocardial ischemia, SGLT2 inhibitors reduce the myocardial infarct size through activation of the signal transducer and activator of transcription 3 and downregulation of inflammatory responses in the infarcted myocardium^3^. However, there is no evidence that SGLT2 inhibitors contribute to the reduction of structural damage to cardiomyocytes during myocardial I/R.

We have previously demonstrated that the cardiac microdialysis technique can monitor myoglobin efflux from cardiomyocytes in the ischemic region of the left ventricle^4^. Sarcolemmal disruption causes myoglobin efflux to the myocardial interstitial spaces. The cardiac microdialysis technique allows us to directly monitor myoglobin efflux into the interstitial spaces. Because there is less blood flow in ischemic regions, diffusion of myoglobin to the plasma should be limited during ischemia. Therefore, the interstitial myoglobin level monitored by cardiac microdialysis technique may serve as a more accurate index of structural damage to cardiomyocytes or cell death than the plasma myoglobin level. In addition, the microdialysis technique is the only method that can locally administer drugs into the ischemic region. To evaluate the effects of SGLT2 inhibitors on myoglobin efflux in the ischemic region, we introduced a microdialysis technique to the heart of anesthetized rats and investigated the effects of acute administration of an SGLT2 inhibitor, dapagliflozin, on myocardial interstitial myoglobin levels in the ischemic region during coronary occlusion followed by reperfusion.

## Methods

### Ethical approval

Animal care was provided in accordance with the Guiding Principles for the Care and Use of Animals in the Field of Physiological Sciences approved by the Physiological Society of Japan and ARRIVE guidelines. The experimental plan was submitted to the Okayama University’s animal experiment committee and approval was obtained (No. OKU-2021480).

### Drug preparation

Dapagliflozin (molecular weight: 408.9) was purchased from Cayman Chemical (Ann Arbor, MI, USA). Dapagliflozin of 5 mg was solved into dimethyl sulfoxide (DMSO, FUJIFILM Wako Pure Chemical Corporation, Osaka, Japan) of 1.223 mL because the solubility was 10 mM in DMSO. This 10 mM solution was divided into 0.1 mL each and stored at − 20 ℃. The solution was diluted by Ringer’s solution of 0.9 mL before the use (1 mM solution). The 1 mM solution of 0.1 mL was diluted by Ringer’s solution of 0.9 mL for the 100 μM solution.

### Animal experiments

Sprague–Dawley rats at the age of 12 weeks (376–427 g) were anesthetized with inhalation anesthesia using 3–4% isoflurane. The rats were intubated after tracheostomies and ventilated with room air mixed with oxygen and 1–1.5% isoflurane. The rats were placed on the heated surgical table to maintain body temperature around 38 ℃. Arterial pressure was monitored by a catheter inserted into the common carotid artery. The catheter was perfused by heparin sodium (5 IU/kg/h) at the speed of 1 mL/h to prevent blood coagulation. No blood transfusion was performed during the experiment. Arterial pressure and heart rate were recorded by a PowerLab Data Acquisition System (ADInstruments, Dunedin, New Zealand). The fourth to sixth ribs on the left side were resected to expose the heart. A 6-0 Prolene polypropylene suture (Ethicon Inc., Somerville, N.J., USA) was passed around the left coronary artery (LCA) for later coronary occlusion. Using a guiding needle, a dialysis probe was implanted in the region perfused by the LCA of the left ventricular wall. In all rats, changes in the color of the ventricular wall during a brief coronary occlusion were examined to confirm whether the dialysis probe was correctly located amid the ischemic region.

**Figure 1:**
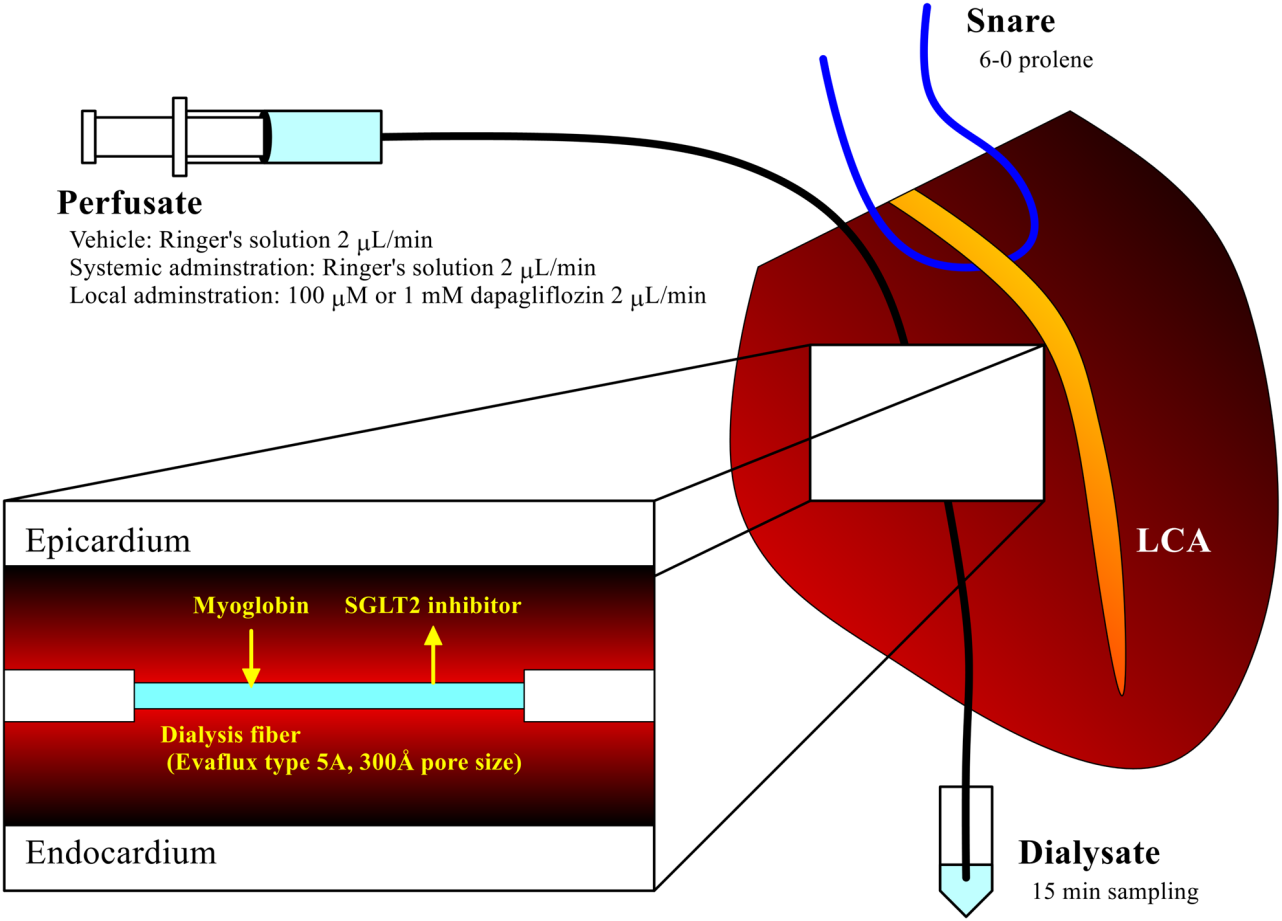
Schema of cardiac microdialysis technique. *LCA* left coronary artery.

The dialysis probe consisted of a dialysis fiber (8 mm length, 0.215 mm o.d., and 0.175 mm i.d. 300 Å pore size; Evaflux type 5A; Kuraray Medical, Tokyo, Japan) and polyethylene tubes glued to both ends of a dialysis fiber. The dialysis probe was perfused with Ringer’s solution and the perfusion speed was set at 2 μL/min (Fig. 1).

Rats were divided into four groups, the vehicle (n = 11), systemic administration (n = 7) and local administration groups (100 μM and 1 mM, n = 12 and 9, respectively). In the vehicle group, the dialysis probe was perfused with Ringer’s solution and no intravenous injection was performed. In the systemic administration group, the dose of dapagliflozin was decided referring to the human daily dose (5 mg/50 kg/day = 100 μg/kg/day). Therefore, dapagliflozin of 40 μg at the concentration of 100 μM and the volume of 1 mL was intravenously injected 30 min before coronary occlusion while the dialysis probe was perfused with Ringer’s solution. In the local administration groups, dapagliflozin of 100 μM and 1 mM was tested. Dapagliflozin of 100 μM or 1 mM was continuously infused through the dialysis probe at least 30 min before ischemia and no intravenous injection was performed.

In each group, after the 15-min of baseline dialysate sampling, a 30-min LCA occlusion followed by 30-min reperfusion was performed while dialysate samples were collected every 15-min. When ventricular fibrillation occurred during myocardial I/R, the left ventricular free wall was tapped using a cotton swab for defibrillation. The numbers of rats alive throughout the experiments were 7, 6, 7 and 7 in the vehicle, systemic and local administration groups of 100 μM and 1 mM, respectively, and these rats were included into the analysis.

At postmortem examination, the excised heart from the euthanized rat was transversely sliced into three or four pieces. The left ventricular cavity was macroscopically examined to confirm that the dialysis fiber was not exposed to the left ventricular cavity.

### Myoglobin measurement

Dialysate myoglobin concentration was measured by immunochemistry using a Cobas h232 plus (Roche Diagnostics, Basel, Switzerland) as an index of myocardial interstitial myoglobin level.

### Statistical analysis

All data were presented as mean ± standard error. The differences were compared by the two-way analysis of variance (ANOVA) followed by the post-hoc Bonferroni test. Statistical significance was defined as P < 0.05 (R4.1.2)^5^.

## Results

### Time courses of heart rate during myocardial ischemia and reperfusion periods in each group

The data of heart rate was shown in Table 1. In the two-way ANOVA, no significant effect was observed in the groups. However, a significant effect was confirmed in the time course (P < 10^–15^). There was no significant interaction between the group and time course.

**Table 1:** Heart rate and blood pressure changes during myocardial ischemia/reperfusion.

Group	Baseline	Time after coronary occlusion		Time after reperfusion	
		0–15 min	15–30 min	0–15 min	15–30 min
Vehicle: (n = 7)					
HR (bpm)	251 ± 2	244 ± 2	237 ± 6	235 ± 3**	233 ± 3*
SBP (mmHg)	116 ± 3	108 ± 2*	106 ± 4	110 ± 6	113 ± 5
DBP (mmHg)	84 ± 3	81 ± 2	77 ± 4*	78 ± 5	81 ± 4
MBP (mmHg)	95 ± 3	90 ± 2	87 ± 4	88 ± 5	91 ± 4
Systemic administration: (n = 6)					
HR (bpm)	255 ± 4	251 ± 3	245 ± 5	240 ± 5	236 ± 6
SBP (mmHg)	120 ± 5	124 ± 5	123 ± 2	120 ± 5	116 ± 4
DBP (mmHg)	91 ± 4	91 ± 4	92 ± 3	87 ± 4	85 ± 4
MBP (mmHg)	101 ± 4	102 ± 4	102 ± 3	98 ± 4	95 ± 4
Local administration of 100 μM: (n = 7)					
HR (bpm)	247 ± 2	239 ± 2	238 ± 3	233 ± 3*	230 ± 2**
SBP (mmHg)	116 ± 4	108 ± 4	108 ± 5	109 ± 4	112 ± 6
DBP (mmHg)	89 ± 5	84 ± 5	83 ± 6	84 ± 5	87 ± 5
MBP (mmHg)	98 ± 4	92 ± 4	91 ± 5*	92 ± 5	95 ± 5
Local administration of 1 mM: (n = 7)					
HR (bpm)	250 ± 2	240 ± 1*	236 ± 2**	233 ± 2**	232 ± 2**
SBP (mmHg)	117 ± 3	110 ± 3*	106 ± 3*	110 ± 2	112 ± 3
DBP (mmHg)	92 ± 3	86 ± 4	81 ± 3*^†^	84 ± 3	86 ± 3
MBP (mmHg)	100 ± 3	94 ± 4	89 ± 3*^†^	93 ± 3	95 ± 3

Data are shown as mean ± standard error.

*HR* heart rate, *SBP* systolic blood pressure, *DBP* diastolic blood pressure, *MBP* mean blood pressure.

*P < 0.05, **P < 0.01 vs. baseline, ^†^P < 0.05 vs. 0–15 min after occlusion.

In the vehicle group, heart rates at 0–15 min and 15–30 min after reperfusion were significantly lower than that at baseline (P < 0.01 and P < 0.05, respectively). In the systemic administration group, the heart rate did not change throughout the experiment. In the local administration group of 100 μM, heart rates at 0–15 min and 15–30 min after reperfusion were significantly lower than that at baseline (P < 0.05 and P < 10^–3^, respectively). In the local administration group of 1 mM, heart rates at 0–15 min and 15–30 min after coronary occlusion and at 0–15 min and 15–30 min after reperfusion was significantly lower than that at baseline (P < 0.05, P < 10^–3^, P < 0.01 and P < 0.01, respectively).

### Time courses of arterial blood pressure during myocardial ischemia and reperfusion periods in each group

The data of systolic, diastolic, and mean blood pressures were shown in Table 1. Two-way ANOVA demonstrated that there was no significant difference in systolic, diastolic, and mean blood pressures among the groups. However, a significant effect was confirmed in the time course (systolic, P < 0.05; diastolic, P < 0.01; mean, P < 0.01). There was no significant interaction between the group and time course.

In the vehicle group, systolic blood pressure at 0–15 min and diastolic blood pressure at 15–30 min after coronary occlusion (P < 0.05 for each) were significantly lower than those at baseline. In the systemic administration groups, systolic, diastolic, and mean blood pressure did not change throughout the experiment. In the local administration groups of 100 μM, mean blood pressure at 15–30 min after coronary occlusion was significantly lower than that at baseline (P < 0.05). In the local administration group of 1 mM, systolic blood pressures at 0–15 min and 15–30 min after coronary occlusion (P < 0.05 for each) were significantly lower than that at baseline. Diastolic and mean blood pressures at 15–30 min after coronary occlusion were significantly lower than those at baseline and 0–15 min after coronary occlusion (P < 0.05 for each).

### Time courses of dialysate myoglobin levels during myocardial ischemia and reperfusion periods

**Figure 2:**
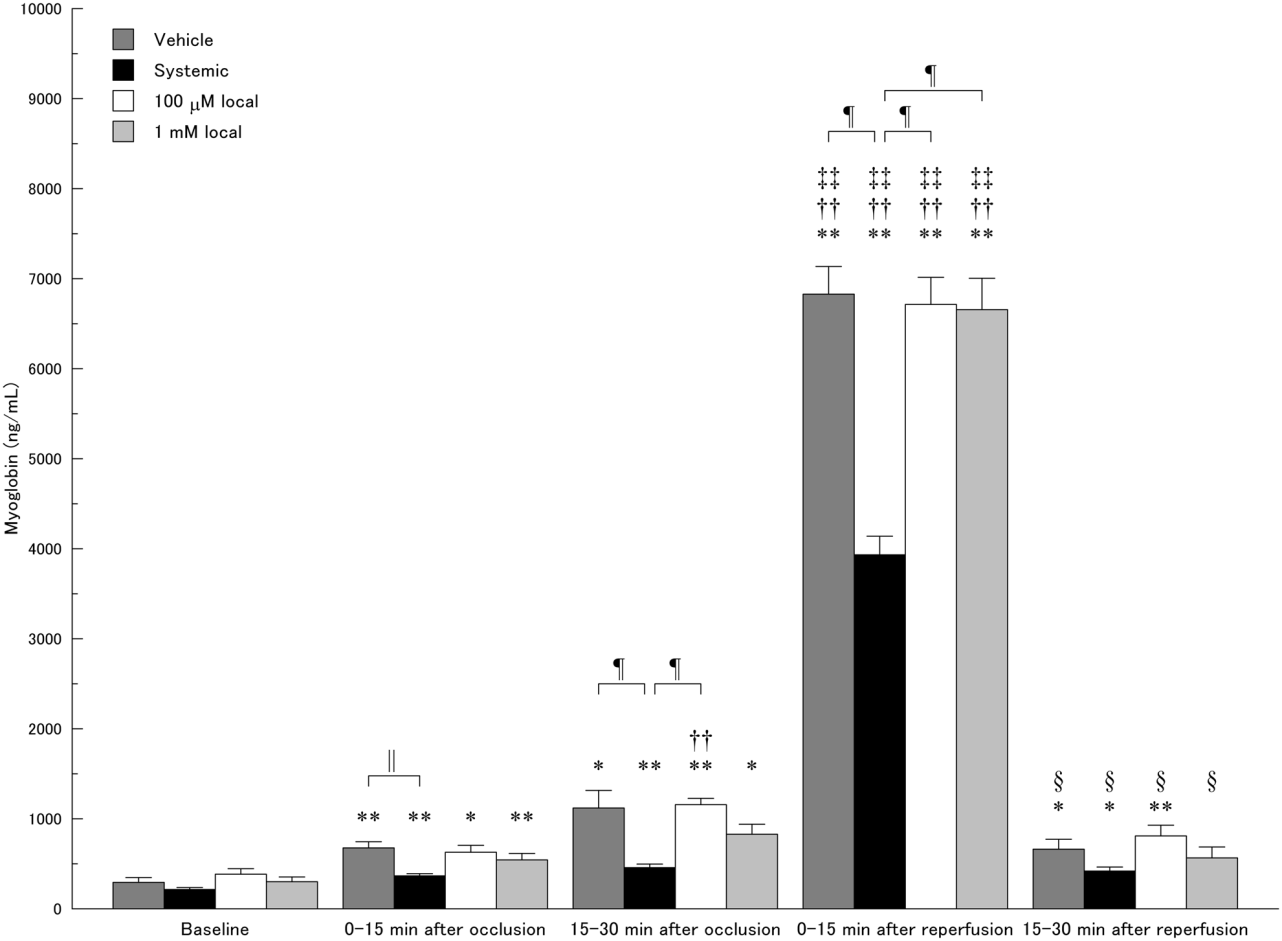
Time course of dialysate myoglobin levels during myocardial ischemia/reperfusion. *P < 0.05, **P < 0.01 vs. baseline; ^††^P < 0.01 vs. 0–15 min after occlusion; ^‡‡^P < 0.01 vs. 15–30 min after occlusion; ^§^P < 0.01 vs. 0–15 min after reperfusion. ^||^P < 0.05; ^¶^P < 0.01.

The data of dialysate myoglobin levels were shown in Fig. 2. A two-way ANOVA confirmed a significant effect in both the group (P < 10^–4^) and the time course (P < 10^–15^). A significant interaction was also confirmed between the group and time course (P < 10^–15^).

In the vehicle group, dialysate myoglobin level significantly increased after coronary occlusion (677 ± 69 ng/mL at 0–15 min (P < 0.01) and 1120 ± 196 ng/mL at 15–30 min after occlusion (P < 0.05 vs. baseline)). Coronary reperfusion further increased dialysate myoglobin level (6829 ± 308 ng/mL at 0-15 min after reperfusion, P < 10^–6^ vs. 15–30 min after occlusion). At 15–30 min after reperfusion, dialysate myoglobin level significantly decreased to 663 ± 110 ng/mL (P < 10^–5^ vs. 0–15 min after reperfusion) but was still higher than that at baseline (P < 0.05).

In the systemic administration group, dialysate myoglobin level significantly increased after coronary occlusion (367 ± 24 ng/mL at 0–15 min (P < 10^–3^) and 458 ± 39 ng/mL at 15–30 min after occlusion (P < 0.01 vs. baseline)). Coronary reperfusion further increased dialysate myoglobin level (3933 ± 206 ng/mL at 0–15 min after reperfusion, P < 10^–3^ vs. at 15–30 min after occlusion). At 15–30 min after reperfusion, dialysate myoglobin level significantly decreased to 420 ± 44 ng/mL (P < 10^–4^ vs. at 0–15 min after reperfusion) but was still higher than that at baseline (P < 0.05).

In the local administration group of 100 μM, dialysate myoglobin level similarly increased after coronary occlusion (629 ± 77 ng/mL at 0–15 min (P < 0.05) and 1157 ± 70 ng/mL at 15–30 min after occlusion (P < 10^–3^ vs. baseline). Coronary reperfusion further increased dialysate myoglobin level (6714 ± 301 ng/mL at 0–15 min after reperfusion, P < 10^–4^ vs. 15–30 min after occlusion). At 15–30 min after reperfusion, dialysate myoglobin level significantly decreased to 810 ± 119 ng/mL (P < 10^–5^ vs. 0–15 min after reperfusion) but was still higher than that at baseline (P < 0.01).

In the local administration group of 1 mM, dialysate myoglobin level significantly increased after coronary occlusion (544 ± 72 ng/mL at 0–15 min (P < 0.01). and 829 ± 111 ng/mL at 15–30 min after occlusion (P < 0.05 vs. baseline). Coronary reperfusion further increased dialysate myoglobin level (6657 ± 348 ng/mL at 0–15 min after reperfusion, P < 10^–5^ vs. 15–30 min after occlusion). At 15–30 min after reperfusion, dialysate myoglobin level significantly decreased to the baseline level.

In the comparison between the groups, there was no significant difference in dialysate myoglobin levels at baseline. At 0–15 min after coronary occlusion, the dialysate myoglobin level in the systemic administration group was significantly lower than that in the vehicle group (P < 0.05). At 15–30 min after coronary occlusion, the dialysate myoglobin level in the systemic administration group was significantly lower than those in the vehicle (P < 0.01) and local administration groups of 100 μM (P < 0.01). At 0–15 min after reperfusion, the dialysate myoglobin level in the systemic administration group was significantly lower than those in the vehicle (P < 10^–5^) and local administration groups of 100 μM and 1 mM (P < 10^–4^ for each). At 15–30 min after reperfusion, there was no significant difference in dialysate myoglobin level between each group.

## Discussion

In this study, we performed the local administration of dapagliflozin to the ischemic region using a cardiac microdialysis technique to investigate the local effect of dapagliflozin on interstitial myoglobin efflux in the ischemic region. The local administration of dapagliflozin did not suppress an increase in dialysate myoglobin concentration during myocardial I/R. When drugs are delivered locally through the dialysis probe, the area over which drugs can be delivered is affected by several factors, including the molecular weight of the drugs and the difference in osmotic pressure between the perfusate and the interstitium. Although the dose of dapagliflozin that passed through the dialysis fiber during the experiment was approximately 72 μg (2 μL/min × 90 min = 180 μL) in the local administration group of 1 mM, this dose did not cause a reduction in myoglobin efflux during myocardial I/R as seen in the systemic administration group. Therefore, the dapagliflozin delivery area may be limited only around the dialysis fiber. Since Sonobe et al. reported that local administration of calpain inhibitors of 500 μM using cardiac microdialysis technique significantly reduced myoglobin efflux after reperfusion^4^, the local effect of dapagliflozin on myoglobin efflux may be small.

**Figure 3:**
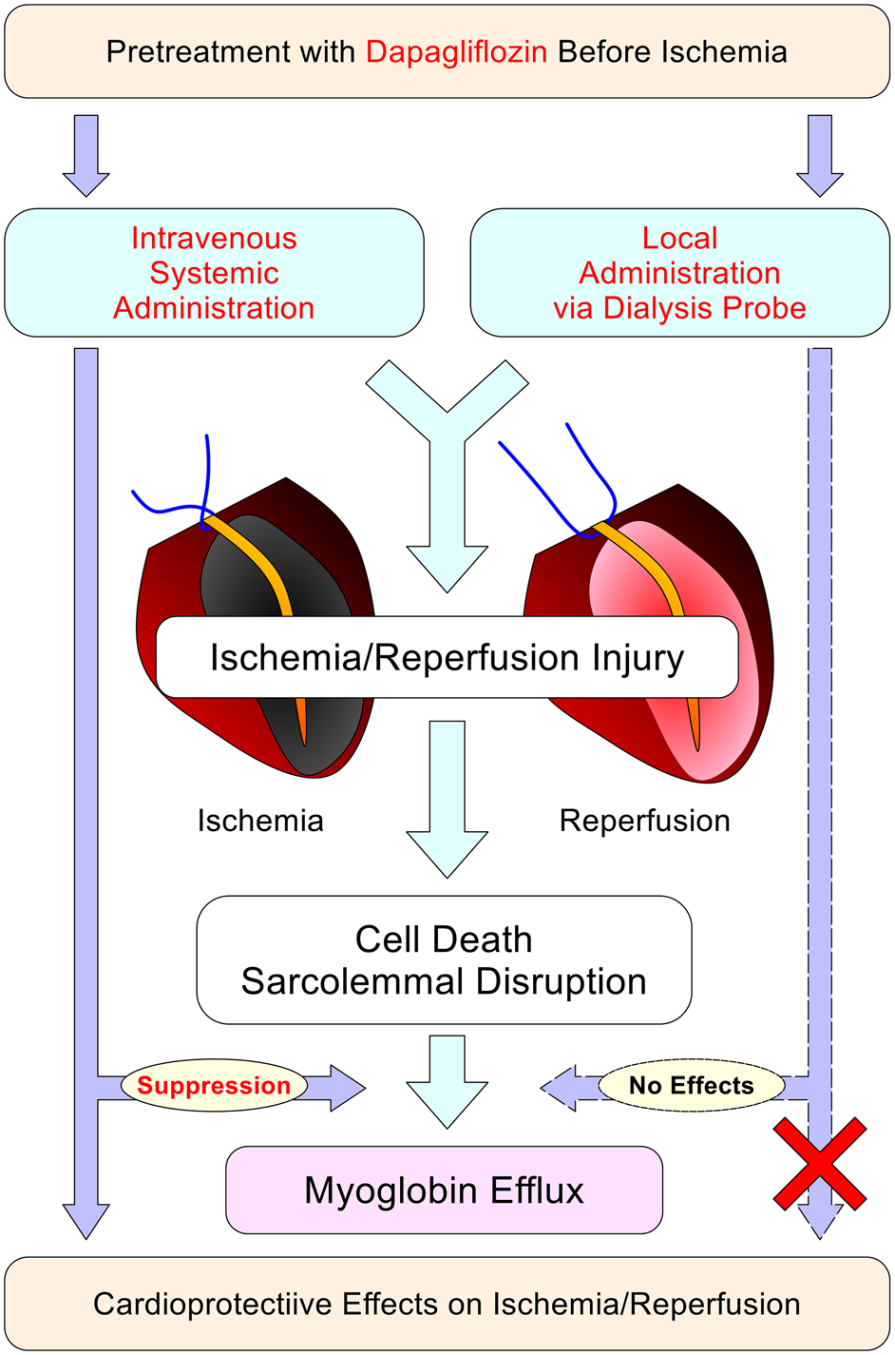
Schematic representation of the results of this study.

On the other hand, systemically administered dapagliflozin before ischemia significantly reduced dialysate myoglobin concentration during myocardial I/R. This result suggests that a systemic mechanism may be involved in the cardioprotective effects of dapagliflozin (Fig. 3). In the systemic administration group, dapagliflozin of 40 μg (= 100 μg/kg/day) was systemically administered to 12-week-old rats, whose average body weight is approximately 400 g, based on the human dosage. Several studies in rats have been reported that oral dapagliflozin at 1 mg/kg/day exerts cardioprotective effects against myocardial I/R^6,7^. Since He et al. have reported that the plasma Cmax of dapagliflozin is 846 μg/L after oral administration of 1 mg/kg in rats^8^, our dose may be high enough compared to these experiments. However, Lahnwong et al. used intravenous dapagliflozin at 1 mg/kg to exert cardioprotective effects in rat models of myocardial I/R^9^. Although a lower dose of dapagliflozin provided sufficient protection in our study, further studies are needed to evaluate the dose-dependent effects of systemic administration.

There are several animal studies focused on the effects of dapagliflozin on myocardial I/R. Lahnwong et al. demonstrated that acute dapagliflozin administration before and during myocardial ischemia exerted cardioprotective effects by attenuating infarct size, increasing ventricular function and reducing arrhythmias^9^. They reported that dapagliflozin administered prior to ischemia provided maximal cardioprotection. On the other hand, Connelly et al. demonstrated that dapagliflozin had no effect on cardiac function after myocardial infarction^10^. Our present study demonstrated that acute administration of dapagliflozin prior to ischemia reduced myoglobin efflux during myocardial I/R. Cardioprotective effects of pre-treated SGLT2 inhibitors against myocardial ischemia have also been reported in the clinical settings. Paolisso et al. reported that diabetic patients treated with SGLT2 inhibitors before acute myocardial infarction had significantly lower in-hospital mortality and lower incidence of arrhythmic events and acute kidney injury after percutaneous coronary intervention^11^. Therefore, to exert the cardioprotective effects of dapagliflozin, acute administration before or during ischemia may be essential.

The mechanism of the cardioprotective effects of dapagliflozin is still under discussion. Hsieh et al. demonstrated that dapagliflozin could mitigate the doxorubicin-elicited cardiotoxicity by reducing oxidative stress, mitochondrial dysfunction, fibrosis, hypertrophy, and inflammation via PI3K/AKT/Nrf2 signaling^12^. Arow et al. also demonstrated that dapagliflozin exerted cardioprotective effects in angiotensin II-stressed diabetic mice by reducing oxygen radicals and the activity of membrane channels related to calcium transport, resulting in decreased fibrosis, reduced inflammation, and improved systolic function^13^.

The antiarrhythmic mechanism of dapagliflozin has been reported in several studies. Dago et al. reported that dapagliflozin as well as empagliflozin increased sodium and inward rectifier potassium currents in healthy human cardiomyocytes^14^. Wu et al. reported that high-dose dapagliflozin reduced ventricular arrhythmias in rats with pulmonary arterial hypertension by improving right ventricular cardiomyocyte Ca^2+^ handling^15^. These mechanisms may contribute to hemodynamic stability during myocardial I/R. However, in our present study, twelve rats died, primarily from ventricular fibrillation and there were no significant differences in the mortality among the groups (P = 0.631 by Fisher’s exact test).

Endothelial effects have also been implicated in the cardioprotective effects of dapagliflozin. Cappetta et al. reported that dapagliflozin reversed coronary endothelial nitric oxide synthase deficit^16^. Uthman et al. reported that dapagliflozin as well as empagliflozin reduced ROS generation and restored NO bioavailability in human coronary endothelium^17^. These effects of SGLT2 inhibitors on the coronary endothelium may increase collateral blood flow to the border zone of the ischemic left ventricle, resulting in cardioprotection against I/R. To exert this cardioprotective effect, systemic administration of SGLT2 inhibitors seems to be necessary. The result of our study may support this hypothesis because only systemic administration of dapagliflozin reduces myoglobin efflux.

There are several limitations in this study. The origin of myoglobin collected by the dialysis probe may be different during ischemia and reperfusion. During ischemia, the movement of high-molecular-weight molecules such as myoglobin may be limited because of the lack of blood flow in the ischemic region. Therefore, myoglobin collected during ischemia may represent structural damage around the dialysis fiber. Reperfusion restores blood flow to the ischemic region and enables movement of high-molecular-weight molecules. Thus, myoglobin collected during reperfusion may represent structural damage not only around the dialysis fiber but also at some distance from the dialysis fiber. In the present experiment, the duration of ischemia was 30 min, and it remains possible that the local effect of dapagliflozin was not exerted within this short period. In addition, isoflurane itself, used as an anesthetic agent, has been reported to have a protective effect against myocardial I/R^18^. Therefore, isoflurane may mask or enhance the inhibitory effect of dapagliflozin on myoglobin efflux. Further investigations on the interaction between isoflurane and dapagliflozin are necessary. Furthermore, we did not perform histological examination and echocardiography in this study. Further investigations are needed to evaluate the pathological effects on the whole heart and the effects on the cardiac function.

## Conclusions

Although SGLT2 is rarely present in cardiomyocytes, acute systemic administration of dapagliflozin prior to ischemia significantly suppressed myocardial myoglobin efflux during myocardial I/R possibly due to a systemic effect. Treatment with dapagliflozin may be helpful in reducing structural damages during myocardial I/R in patients at high risk for acute coronary syndrome.

## Data Availability

The datasets used and analyzed during the current study are available from the corresponding author on reasonable request.

## Abbreviations

ANOVA: Analysis of variance
DMSO: Dimethyl sulfoxide
I/R: Ischemia/reperfusion
LCA: Left coronary artery
SGLT2: Sodium-glucose cotransporter 2
